# Quantum Interference Enhancement of the Spin-Dependent Thermoelectric Response

**DOI:** 10.1021/acsnano.4c01297

**Published:** 2024-04-23

**Authors:** Runa X. Bennett, Joshua R. Hendrickson, Justin P. Bergfield

**Affiliations:** † Department of Physics, Illinois State University, Normal, Illinois 61790, United States; ‡ Air Force Research Laboratory, Sensors Directorate, Wright-Patterson Air Force Base, Dayton, Ohio 45433, United States; § Department of Chemistry, Illinois State University, Normal, Illinois 61790, United States

**Keywords:** quantum spin-thermopower, coherent transport, quantum interference, many-body transport theory, nonequilibrium Green’s functions, single-molecule junction

## Abstract

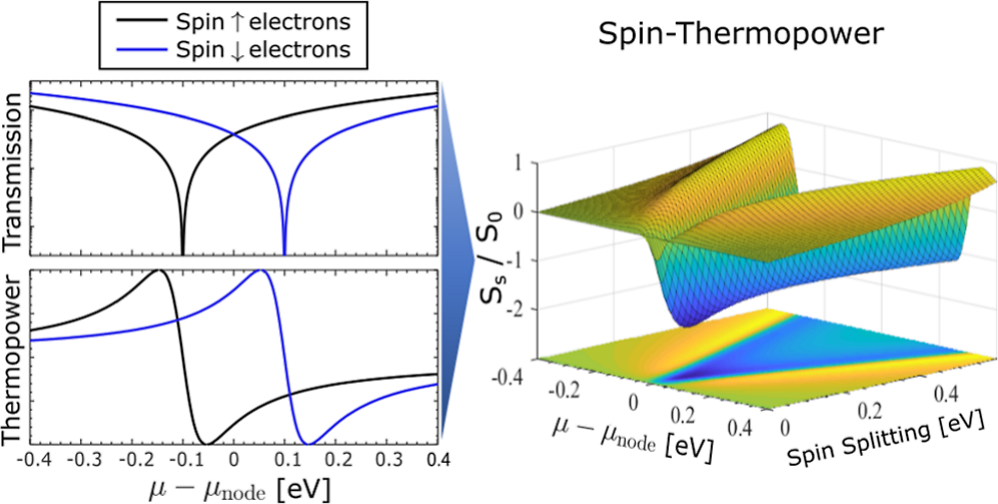

We investigate the influence of quantum interference (QI) and broken spin-symmetry on the thermoelectric response of node-possessing junctions, finding a dramatic enhancement of the spin-thermopower (*S*_s_), figure-of-merit (*Z*_s_*T*), and maximum thermodynamic efficiency (η_s_^max^) caused by destructive QI. Using many-body and single-particle methods, we calculate the response of 1,3-benzenedithiol and cross-conjugated molecule-based junctions subject to an applied magnetic field, finding nearly universal behavior over a range of junction parameters with *S*_s_, *Z*_s_*T*, and reaching peak values of , 1.51, and 28% of Carnot efficiency, respectively. We also find that the quantum-enhanced spin-response is spectrally broad, and the field required to achieve peak efficiency scales with temperature. The influence of off-resonant thermal channels (e.g., phonon heat transport) on this effect is also investigated.

Thermoelectric devices directly convert heat into electrical energy, making them ideal for a host of clean-energy applications. In response to an applied temperature difference, heat, spin, and charge flow through a junction until an induced potential is formed and equilibrium is reached. The ratio of induced thermoelectric voltage to applied temperature gradient is known as the thermopower (Seebeck coefficient) and is both an important measure of a device’s performance and underlying transport processes.^1−4^

In junctions with dimensions commensurate with or less than the de Broglie wavelength of the charge carriers, coherent wave effects can dominate the transport. Of particular interest are destructive quantum interference (QI) features, known as nodes, which occur when all transmission amplitudes cancel completely. Nodes, which emerge from the interplay between quantum coherence and the symmetries of the Hamiltonian, can often be understood in terms of a junction’s chemical structure using single-particle concepts,^5−9^ although this is not always the case.^10−13^ Since nodes stem from structure, they are not generally described by single-level or quantum dot models. In the vicinity of a node, the entropy and charge currents are reduced differentially. Their ratio, the entropy per unit charge, is the thermopower and is predicted to exhibit an enhancement due to destructive QI.^14,15^

The performance of a device can also be enhanced by QI effects.^15,16^ A common metric for evaluating device performance is *ZT* = *S*^2^*GT*/κ, where *S*, *G*, κ, and *T* represent thermopower, electrical conductivity, total thermal conductivity, and temperature, respectively. Traditional approaches to increase *ZT* are often derived from bulk material concepts,^17^ which may neglect the dual nature of quantum excitations, potentially missing key effects. For instance, large violations of the Wiedemann–Franz law are predicted to occur near transmission nodes owing to the breakdown of the free-electron model of conduction.^14^ This breakdown is of fundamental interest and is predicted to give rise to *ZT* and thermodynamic efficiency enhancements which are spectrally broad and scalable.^15^

When time-reversal symmetry is broken (e.g., in systems operating far from equilibrium, in certain chiral systems, or via the application of an external magnetic field), a spin-voltage may be generated in response to a temperature gradient via the spin-dependent Seebeck effect.^18^ The corresponding spin-dependent thermopower, which can be measured, e.g., via the inverse spin-Hall effect,^19^ has been investigated in a variety of ferromagnetic,^19^ graphene and nanoribbon,^20−24^ single and multilevel quantum dot models,^25−34^ molecular magnets,^35−40^ and chiral systems,^41^ and quantifies the coupling between heat and spin degrees of freedom.

We focus on the response of single-molecule junctions (SMJs), open quantum systems composed of a small organic molecule coupled to nonmagnetic electrodes. The electronic transport in these systems is predominantly quantum coherent and elastic, even at room temperature and in noisy chemical and electromagnetic environments,^4,42−44^ and small molecules naturally possess symmetries which harness QI effects.^5−7^ Organic molecules are also composed of light elements with weak spin–orbit coupling, ensuring the independence of each spin-channel’s transport. Recent thermopower measurements in molecule-based junctions^45^ support the prediction of an enhanced thermoelectric response near a node,^14^ motivating our investigation of a spin-dependent analog.

In this paper, we derive expressions for spin-dependent transport through an interacting nanostructure and apply them to investigate the influence of QI and broken spin-symmetry on the thermoelectric and thermodynamic response of node-possessing SMJs. Employing both many-body^46^ and single-particle methods, we calculate the transport through two distinct SMJs, a benzene-based junction and a cross-conjugated molecule-based junction, finding that near a node their responses are nearly independent of molecular structure, treatment of correlations, and electrode-molecule coupling, while their transmission functions differ by orders of magnitude.

With the application of an external magnetic field, the node in each spin’s transmission function shifts, giving rise to a quantum enhancement of the spin-thermopower (*S*_s_), spin figure-of-merit (*Z*_s_*T*), and the maximum spin thermodynamic efficiency (η_s_^max^). For junctions with a single-quadratic node, these quantities are enhanced over a wide range of field strengths and electrode-molecule alignment values, reaching maxima of *S*_s_=  (∼313 μV/K at room temperature), *Z*_s_*T* = 1.51, and η_s_^max^ = 28% of Carnot efficiency. Although these values are reduced by additional off-resonant thermal channels (e.g., stemming from phonons), the charge analogs of these quantities scale superlinearly with the order of the interference feature,^15^ indicating the potential of molecule-based spin-caloritronic devices.

## Spin-Dependent Thermoelectric Theory

In linear response, the elastic contribution to the total charge current *I*, total spin current *I*_s_, total heat current *Q*, and spin heat current *Q*_s_ flowing in a two-terminal junction may be expressed as
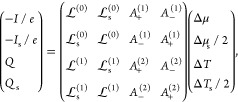
1where  are the νth order total and spin Onsager (kinetic) functions and, to simplify the notation, we introduced
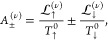
2where *T*_σ_^0^ is the equilibrium temperature of the electrode’s σ-spin electrons. The currents are driven by the appropriate charge and spin chemical potential differences and temperature gradients given by^18,28,47^
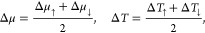
3
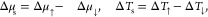
4where Δμ_σ_ = μ_σ_^*L*^ – μ_σ_^*R*^ and Δ*T*_σ_ = *T*_σ_^*L*^ – *T*_σ_^*R*^ are the spin-voltage and spin-temperature differences between the left and right-hand electrodes, respectively. Eq 1 is not limited to quantum systems or systems with weak interparticle interactions, i.e., it is also applicable to strongly correlated systems. Nonlinear terms arising, for instance, from inelastic contributions to the transport are typically weak in SMJs composed of small organic molecules like the ones considered here.^1^

Under the influence of an applied temperature gradient, charge carriers drift in accordance with the second law of thermodynamics until an induced thermoelectric voltage compensates and equilibrium is reached. The ratio between the induced voltage (spin-voltage) and the applied temperature difference is called the thermopower (spin-thermopower). Starting from eq 1, the charge and pure spin-thermopower may be expressed as
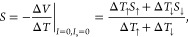
5and
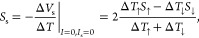
6respectively, where the spin σ electron’s thermopower is given by
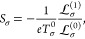
7and Δ*V*_(s)_ = −Δμ_(s)_/*e* are the total charge (spin) thermoelectric voltages.

Although the equilibration between spin distributions can be inhibited by reduced dimensionality and weak inelastic scattering,^48,49^ equilibrium is rapid in the nonmagnetic bulk electrodes. Therefore, in the remainder of this article, the macroscopic electrodes are assumed to have spin-independent temperatures such that Δ*T*_σ_ ≡ Δ*T* and *T*_σ_^0^ ≡ *T*.

The dimensionless figure-of-merit *ZT* and spin figure-of-merit *Z*_s_*T* are often used to quantify a device’s performance and may be defined as^50,51^
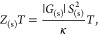
8where *G* and *G*_s_ are the total and spin electrical conductances, respectively, and κ is the total thermal conductance. For a high-performance device, *G*_(s)_ and *S*_(s)_ should be large to maximize the charge (spin) current flowing and the conversion between heat and charge (spin) degrees of freedom, respectively, while the thermal conductance κ should be minimized so as to maintain the thermal gradient across the device.

In terms of Onsager functions, the total charge (spin) electrical conductance is given by
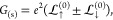
9and the electronic contribution to the thermal conductance is given by
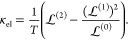
10The total thermal conductance κ = κ_el_ + κ_alt_ is a combination of electronic and alternate thermal transport channels (e.g., stemming from phonons or blackbody radiation). In SMJs composed of small organic molecules, the dominant contribution to κ_alt_ is typically from phonons, which can have thermal conductivity values of tens of pW/K range at room temperature,^52−54^ although these values are highly sensitive to chemical composition.

Thermodynamically, a device’s performance is characterized by the efficiency η: the ratio of useable work to input heat. In general, η depends on molecular structure, energy alignments, applied temperature differences and voltages, etc.^15^ However, the maximum total and spin thermodynamic efficiency can be found by optimizing η with respect to linear-response quantities giving^55^
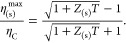
11

We focus on the transport through SMJs composed of a small organic molecule coupled to nonmagnetic macroscopic electrodes. The total Hamiltonian of this system may be expressed as *H* = *H*_mol_ + ∑_α=*L*,*R*_*H*_lead_^(α)^ + *H*_t_^(α)^, where
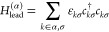
12describes the conduction electrons in the (nonmagnetic) lead α, and
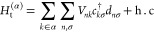
13describes the tunneling of electrons between lead α and the molecule.

The effective molecular Hamiltonian for the π-system was derived from first principles, starting with Schrödinger’s equation for the full interacting junction using a renormalization procedure which includes off-resonant degrees of freedom (e.g., from the σ-system, image charge effects, etc.) implicitly as renormalized energy and coupling terms.^56^

In a basis of localized orbitals, the Hamiltonian derived using this method may be written as^56^
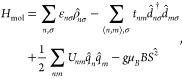
14where ε_*n*σ_ is the effective on-site potential for σ-spin electrons on orbital *n* of the molecule,  creates (annihilates) σ-spin electrons on orbital *n*, ,  is the net charge operator in units of –*e*, and *t*_*nm*_ are the effective tight-binding matrix elements. The Coulomb interaction *U*_*nm*_ between electrons in the *n*th and *m*th orbitals is found via multipole expansion such that^56^
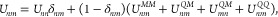
15where *U*^*MM*^, *U*^*QM*^, and *U*^*QQ*^ are the monopole–monopole, quadrupole–monopole, and quadrupole–quadrupole interactions between renormalized π-orbitals, respectively. These interactions are assumed to be screened by a uniform dielectric ϵ, e.g., arising from the σ-electrons. Time-reversal symmetry is broken by the external magnetic field  via the Zeeman term, where μ_B_ is the Bohr magneton, *g* is the Landé factor, and  is the z-component of the total spin operator. With a Zeeman term, all molecular energies (and therefore nodes) shift with the application of an external field.

Although this Hamiltonian is formally similar to certain semiempirical methods (e.g., the Pariser–Parr–Pople (PPP) or “extended Hubbard” methods),^57−59^ this is an ab initio theory which accurately describes arbitrary π-electron systems.^56^ Moreover, the π-electron quadrupole moment *Q* is a physical quantity which takes the place of the ad hoc interaction parametrizations used in other models. Additional details regarding this theory are included in the Methods section.

The room-temperature transport through an SMJ composed of a small organic molecule is primarily coherent and elastic. This allows us to express the Onsager functions as
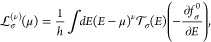
16where  is the Fermi–Dirac distribution with spin-dependent chemical potential μ_σ_^0^ and temperature *T*_σ_^0^. The transmission probability for an electron with energy *E* and spin σ is given by
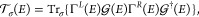
17where  is the junction’s retarded Green’s function, Γ^α^ are the tunneling-width matrices, and the subscript in the trace indicates a sum over spin subspace. In systems composed of heavier elements where spin–orbit coupling cannot be neglected, spin would no longer be a good quantum number and an external field would mix the spin-channel’s transport.

Many-body calculations were performed using the molecular Dyson equation (MDE) theory,^46^ a nonequilibrium Green’s function (NEGF)-based theory which includes all molecular excited and charge states as well as their associated multiparticle matrix elements. Effective single-particle transport calculations utilize the same framework with *U*_*nm*_ = 0, which is equivalent to an effective Hückel electronic structure description (see Methods). More sophisticated effective single-particle theories, such as density functional theory (DFT) with currently known exchange–correlation functionals, give qualitatively similar results for the systems treated here.^12,60−64^

## Results and Discussion

We focus on room-temperature transport through SMJs composed of small organic molecules whose primary conducting channel has a node far detuned from any molecular addition or removal energy. In this regime, the low-energy transport is dominated by the HOMO and LUMO resonances, whose tails give rise to a quadratic node in that channel.^6,9−11,65,66^

**Figure 1:**
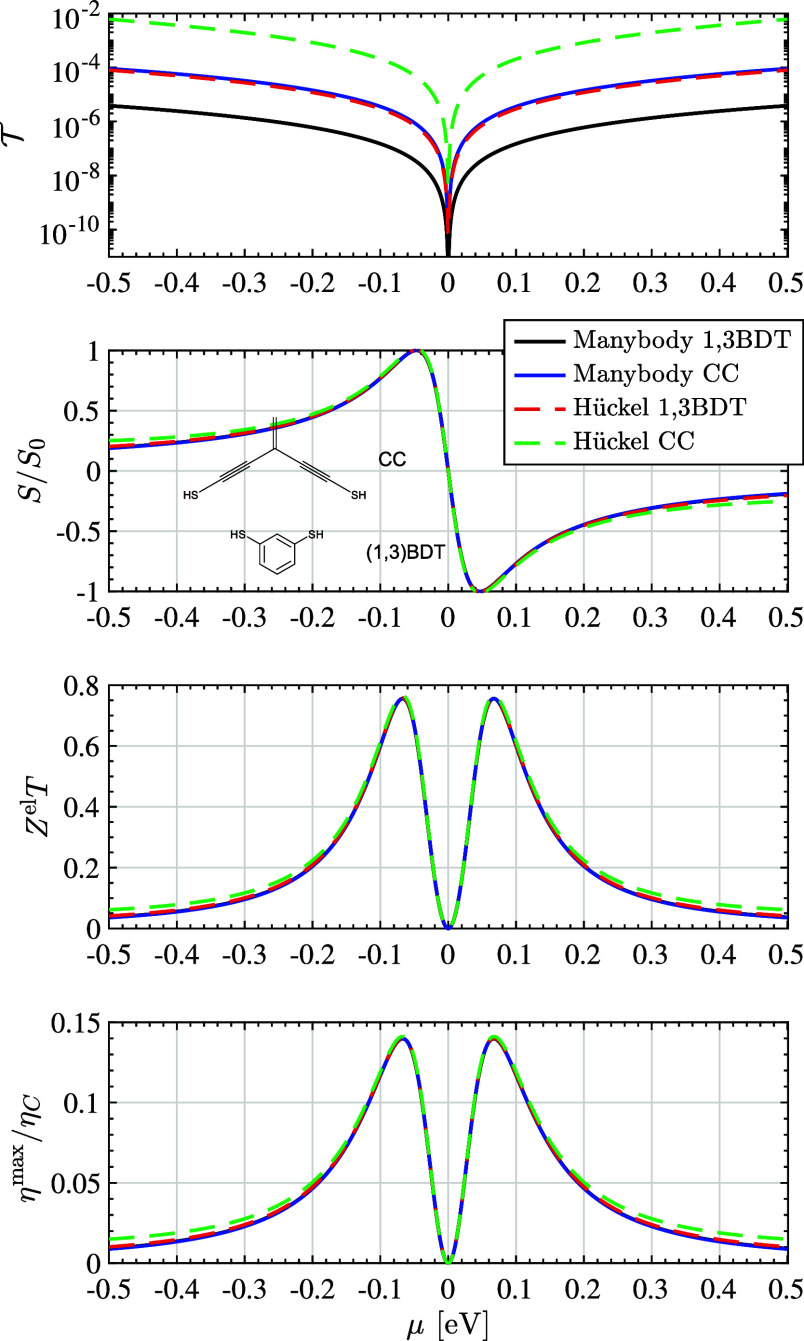
The total transmission , thermopower *S*, electronic figure-of-merit *Z*^el^*T*, and peak thermodynamic efficiency η^max^ of 1,3-BDT and CC junctions are shown calculated using many-body (MDE) and single-particle (Hückel) theories near the node. Although  differ by orders of magnitude, *S*, *Z*^el^*T*, and η^max^ are insensitive to junction details and electronic structure methods used in these cases away from resonance. All spectra are aligned to their respective nodal energies (set here to μ = 0). Calculations are for *T* ≡ *T*_σ_^0^ = 300 K in the absence of a magnetic field such that  156 μV/K.

For illustration, we consider the transport through Au-3-methylenepenta-1,4-diyne-1,5-dithiol-Au (CC) and Au-1,3-benzenedithiol-Au (1,3-BDT) junctions, whose bridging molecules are shown schematically in the second panel of Figure 1. The parameters and geometries used can be found in the Methods section. Both the cross-conjugated CC and conjugated 1,3-BDT junctions are predicted to exhibit a quadratic node in the midgap region of their π-system transmission spectrum,^12,14,62,64,65,67−69^ although the nodal energies in each junction differ (see Supporting Information). The σ-system is only included implicitly via π-EFT renormalization since the transport spectrum of the σ channel is nearly featureless near the nodal energy in these junctions.^62,64,67^

The calculated response of each junction near the nodal energy (set to μ = 0 for each spectrum independently) is shown as a function of the electrodes’ chemical potential μ in Figure 1, using both many-body and single-particle theories. As expected, the total transmission , shown in the top panel, varies significantly depending on the chemical structure of the junction and theoretical method used. In contrast, the thermopower *S* near the node—which is proportional to the logarithmic derivative of (70)—is nearly indistinguishable in each case, reaching a peak value of *S*_0_ =  when .^14^ Similarly, *Z*^el^*T* and η^max^, shown in the bottom two panels, also exhibit similar behavior near the node, reaching peak values of 0.76 and 13.98% of Carnot, respectively, in each case when |μ| = π*kT*(7/15)^1/4^.^15^

In the vicinity of nodes stemming from detuned molecular resonances, the (spin) thermoelectric response is nearly independent of chemical composition, meaning that our results apply to a wide variety of physical systems. However, this universality does not extend over the entire spectrum. For instance, the response near the HOMO and LUMO resonances does strongly depend on the junction details, including the coupling strength and asymmetry (see Supporting Information). Other systems may exhibit nodes, e.g., those stemming from Fano antiresonances,^5,9,71^ whose thermoelectric enhancement and scaling strongly depend on the chemical composition or electronic structure method used. For consistency, we utilize many-body theory calculations for the remainder of this article; effective Hückel + NEGF calculations can be found in the Supporting Information.

**Figure 2:**
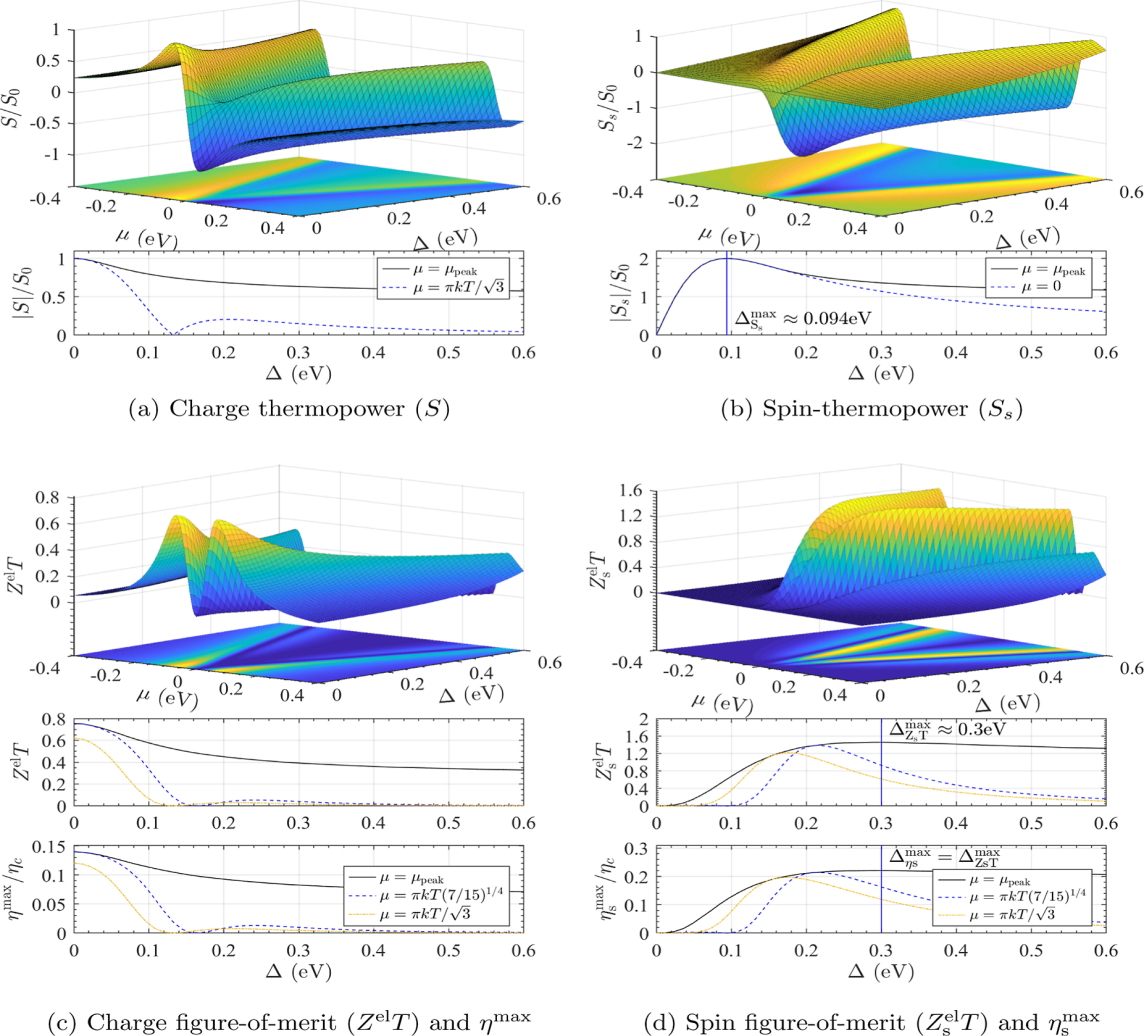
Calculated (a) charge thermopower *S*, (b) spin-thermopower *S*_s_, (c) *Z*^el^*T*, η^max^ and (d) spin *Z*_s_^el^*T*, η_s_^max^ spectra of a 1,3-BDT junction’s π-system shown as functions of electrode chemical potential μ and spin-splitting Δ = 2*g*μ_B_*B* using many-body theory. Charge quantities decrease monotonically with increasing Δ. Spin quantities are peaked, reaching maxima when  and Δ ≈ 11.8*kT* for *S*_s_ and *Z*_s_*T* (η_s_^max^), respectively. In the lower panels, quantities are shown with μ tuned to give the maximum response (μ = μ_peak_) and with μ fixed to specific values. Calculations are for *T* = 300 K such that  156 μV/K.

When time-reversal symmetry is broken via the application of magnetic field *B*, the spin-degenerate transmission node is separated into two nodes split by energy Δ = 2*g*μ_B_*B*. The calculated thermopower *S*, spin-thermopower *S*_s_, *Z*^el^*T* (η^max^), and *Z*_s_^el^*T* (η_s_^max^) spectra of a 1,3-BDT junction are shown as a function of chemical potential μ and spin-splitting Δ in Figure 2. In the lower panels of each subsection, cross sections of each quantity are shown with μ tuned to maximize that quantity (i.e., μ = μ_peak_) and with μ fixed to certain zero-splitting peak values. Although spin–orbit coupling is neglected, μ_peak_ is not simply the zero-field peak shifted by ±Δ/2 due to the energy dependence of each quantity.

As shown in subfigure (a), the charge thermopower magnitude |*S*| is largest when Δ = Δ_*S*_max = 0 and decreases monotonically as each spin-channel becomes increasingly hybridized by the external field. In contrast, |*S*_s_|, shown in subfigure (b), has a peaked structure. Assuming spin-independent electrode temperatures, *S*_s_ = *S*_↑_ – *S*_↓_ and |*S*_s_| is maximized when the peak of *S*_↑_ aligns with the minimum of *S*_↓_, or vice versa. In the case of a single quadratic node, max(*S*_s_) = 2*S*_0_ when μ = 0 and , where
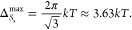
18Similarly, *Z*^el^*T* and η^max^ decrease monotonically as a function of Δ while *Z*_s_^el^*T* and η_s_^max^ are peaked, as indicated in subfigures (c) and (d), respectively. *Z*_s_^el^*T* reaches a maximum value of 1.51 while η_s_^max^ reaches 27.96% of Carnot when , where
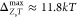
19and μ ≈ 4.26*kT*. We use numerical methods to determine these relationships (Supporting Information). The peak values of the spin quantities are twice their charge analogs owing to the spin voltage defined in eq 4. In all cases, variations in the η^max^ and *Z*^el^*T* spectra follow one another, indicating that *Z*_(s)_^el^*T* is a good measure of the maximum (spin) thermodynamic device performance in these systems.

Near or below the Kondo temperature *T*_K_, the thermopower^72^ and spin-thermopower^30,47,73^ can exhibit sign-changes and other interesting effects stemming from the formation of Kondo correlations. We have neglected these effects in our treatment of transport, limiting our results to temperatures above *T*_K_, which for the CC and 1,3-BDT junctions considered here correspond to ∼298 and ∼6 μK, respectively (see Supporting Information).

Through careful selection of electrode materials and molecular species,^74^ as well as by employing various gating techniques,^75,76^ shifts of μ by ∼eV have been realized in SMJs.^77−79^ External fields of ∼1 T have been applied to organic-based SMJs^80^ and fields of up to several Tesla have been applied to junctions based on heavier elements, resulting in spin splittings on the order of meV.^81^ For the junctions considered here, |*S*_s_| is maximized at 1 K when Δ = 0.313 meV, corresponding to an external field of 1.35 T, close to what has been realized experimentally.^80^

Despite the substantial field required to achieve peak performance, the QI effects on *S*_(s)_ and *Z*_(s)_^el^*T* are spectrally broad and significant enhancement is predicted for a wide range of μ and Δ values. However, as indicated in the dashed lines in each panel of Figure 2, these quantities decrease more rapidly from their maxima as Δ (or μ) is varied when μ (or Δ) is fixed rather than tuned, a trend that becomes more pronounced when additional thermal channels are introduced. Also, the thermopower and figure-of-merit peak at different values, indicating devices designed to harness QI effects, should be concordant with the application.

### Additional Thermal Transport Channels

The discussion presented above focused on the response of the nodal transport channel. Often, alternate thermal transport paths (e.g., those stemming from phonons or blackbody radiation) are present which reduce the QI enhancement as
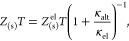
20where *Z*_(s)_^el^*T* is the nodal-channel’s contribution to the (spin) figure-of-merit and κ_alt_ is the thermal conductance of the alternate channel.

**Figure 3:**
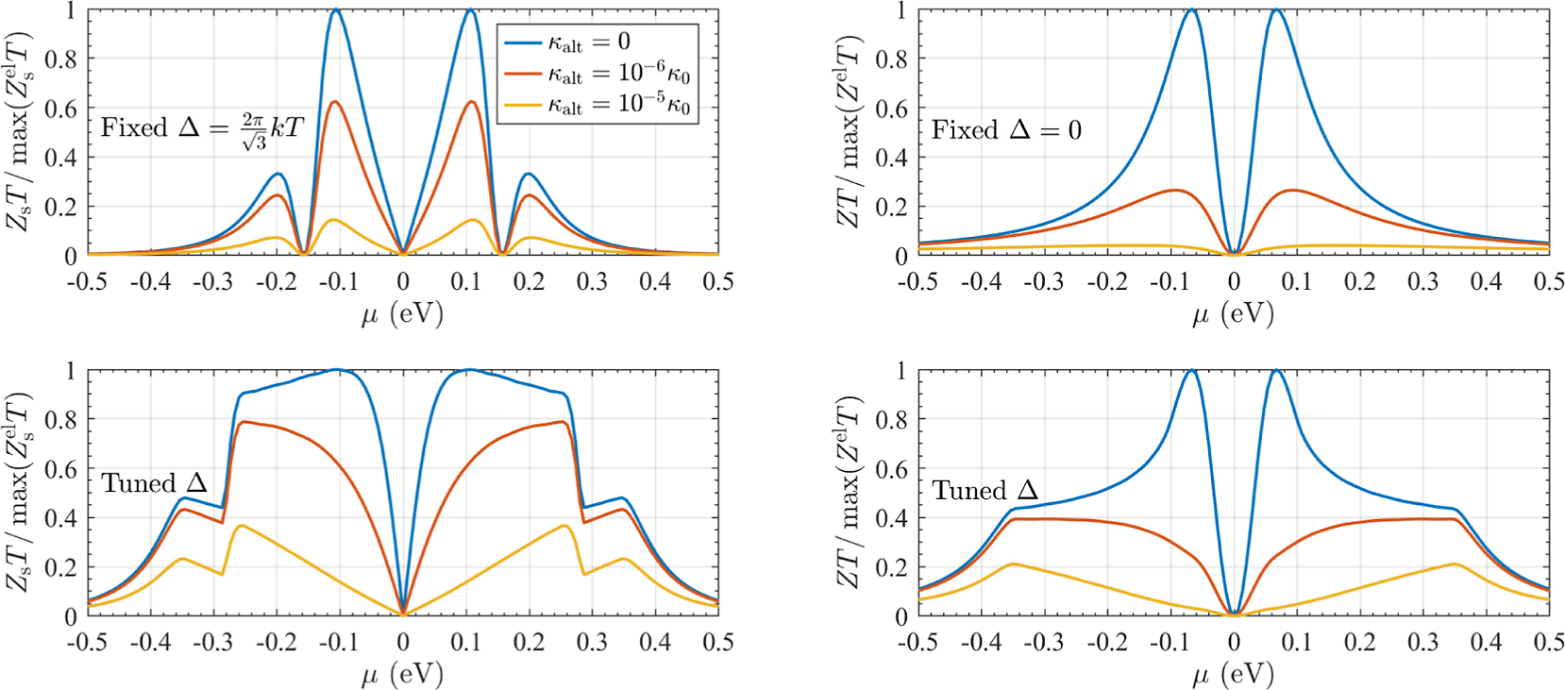
Influence of κ_alt_ on the *Z*_s_*T* (left panels) and *ZT* (right panels) spectra of a 1,3-BDT junction calculated using many-body theory. Values are normalized to the node-possessing channel’s peak value. Owing to the influence of Δ on κ, *Z*_s_*T* is less sensitive to additional thermal channels than *ZT*. When κ_alt_ = 10^–6^κ_0_, *Z*_s_*T* is reduced by 21.1% when Δ is tuned and 37.4% when Δ is fixed while *ZT* is reduced by 60.6 and 73.5% when tuned and fixed, respectively. The optimal Δ is a function of the total transport, so the importance of tuning Δ increases with κ_alt_. When κ_alt_ = 10^–5^κ_0_, *Z*_s_*T* is reduced by 63.3 and 85.6% when Δ is tuned or fixed, respectively, while *ZT* is reduced by 79 and 96%, respectively. κ_0_ = π^2^*k*^2^*T*/3*h* ∼ 289 pW/K at *T* = 300 K.

The influence of κ_alt_ on *Z*_s_*T* and *ZT* is shown for a 1,3-BDT junction with both fixed and tuned Δ in Figure 3. Comparing the left- and right-hand panels of the figure reveals that for the same κ_alt_, the peak values of *Z*_s_*T* are reduced (proportionally) less than those of *ZT*. For the systems considered here, this occurs because *Z*_s_*T* peaks when Δ > 0 which increases *κ*_el_ and reduces κ_alt_/κ_el_ in eq 20. For instance, when κ_alt_ = 10^–6^κ_0_ and Δ is fixed (top panels), the peak value of *Z*_s_*T* is reduced by 37.4% from the nodal spectrum’s peak value while the peak value of *ZT* is reduced by 73.5%.

When Δ is tuned, the reductions generally decrease, and the spectral width of the enhancement increases. For the same κ_alt_ value with tuned Δ (bottom panels), the reduction of the peak value of *Z*_s_*T* becomes 21.1% while *ZT* is reduced by 60.6% of the nodal values, although in both cases the enhancement has broadened significantly in μ.

As κ_alt_ is increased, so does the importance of tuning Δ. With κ_alt_ = 10^–5^κ_0_, *Z*_s_*T* is reduced by 85.6% when Δ is fixed but only 63.3% when Δ is tuned. Similarly, *ZT* is reduced by 96% when Δ is fixed and 79% when Δ is tuned for the same κ_alt_. The η_(s)_max spectra qualitatively match the *Z*_(s)_*T* spectra. An analysis of their correspondence and an evaluation of the importance of many-body correlations for these quantities can be found in the Supporting Information.

The range of κ_alt_ was selected to demonstrate the influence of additional thermal channels on the performance of a molecular spin-caloritronic device; larger values will further reduce the device performance. The relevant magnitude depends on κ_el_ in the vicinity of the node (cf. Figure 1, where at low-temperature ) which itself depends critically on the specific chemical composition of the SMJ. In molecular junctions, these values can be adjusted over a broad range through electrode material choice and rational chemical design,^53,82−86^ highlighting the importance of tailoring molecular structure to optimize device performance and the potential of molecule-based devices to realize efficient spin-caloritronic materials.

## Conclusions

We find that QI can enhance the spin-dependent thermoelectric and thermodynamic responses of node-possessing junctions. In systems with weak spin–orbit coupling, the application of an external magnetic field causes the node in each spin-channel’s transmission function to shift, giving rise to peaks in the spin-dependent thermoelectric response.

The organic-based SMJs considered here have quadratic nodes in their π-system, leading to peak values for *S*_s_, *Z*_s_*T*, and η_s_^max^ of 3.6*k*/*e* (∼313 μV/K at room temperature), 1.51, and 28% of Carnot efficiency, respectively, for optimal field strengths and electrode-molecule level alignments. These values are reduced by additional thermal channels (e.g., stemming from phonons); however, the charge analogs of these quantities are predicted to scale with node order,^15^ signifying the potential of molecule-based spin-caloritronic devices.

Despite the large field needed to realize peak response at room temperature, the QI-enhanced response is broad, and the spin-splitting required to realize splitting needs scales linearly with temperature. In addition, alternative symmetry-breaking mechanisms, e.g., those arising from molecular^87^ or junction^88^ symmetries, nonequilibrium conditions, or exchange fields,^89−91^ may reduce or eliminate the need for such large fields entirely.

Our results are not limited to SMJs; however, the thermoelectric response of junctions composed of small molecules is dominated by quantum effects even at room temperature. In addition, molecular structure and junction symmetry can be used to control these effects in the transport, which, in turn, can be engineered with atomic precision using the techniques of chemical synthesis, making them attractive for a wide range of applications.

## Methods

### Molecular Hamiltonian

The Hamiltonians for the benzenedithiol and CC molecules considered in this article were derived from first principles using the effective field theory for the π-system (π-EFT) developed in ref (56), which we briefly outline here. Within the Born–Oppenheimer approximation, the electronic Hamiltonian for an isolated molecule be written as
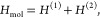
21where the one-body term is given by
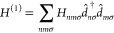
22with
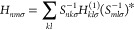
23and
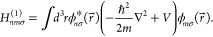
24*S*_*nm*_ = ⟨ϕ_*n*_|ϕ_*m*_⟩ is the overlap matrix element between atomic orbitals ϕ_*n*,*m*_. In the main text, we set ε_*n*σ_ = *H*_*nn*σ_^(1)^ and , for conciseness.

The two-body term may be written in terms of the atomic orbital basis as
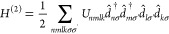
25where
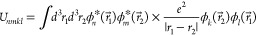
26and
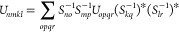
27The effective Hamiltonian for the π-electron system is then found by excluding orbitals which do not participate in bonding and expanding the interaction matrix in powers of the interatomic bond length. This allows us to reduce the interaction matrix as *U*_*nm*_ = δ_*nl*_δ_*mk*_*U*_*nmkl*_ via the renormalization of the atomic orbitals.^56^ Note this is mathematically equivalent to the neglect of differential overlap approximation,^92−94^ although the only requirement in π-EFT is that the effective Hamiltonian be local.^56^

Expanding in powers of interatomic bond length leads to the multipole expansion
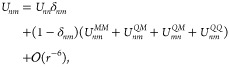
28where *U*_*nm*_^*MM*^ is the monopole–monopole interaction, *U*_*nm*_^*QM*^ is the quadrupole–monopole interaction, and *U*_*nm*_^*QQ*^ is the quadrupole–quadrupole interaction between renormalized π-orbitals, given explicitly in ref (56). After imposing particle-hole symmetry on the isolated molecule, a symmetry which is justified by the success of other (e.g., PPP-type^57,95^) semiempirical methods, and adding a Zeeman term we arrive at the effective molecular Hamiltonian used in the main text.

The π-EFT parameters were determined via a renormalization procedure in which experimental data were fit to experimental quantities that should be accurately reproduced by a π-electron-only model. In particular, we simultaneously optimized predictions of (1) the vertical ionization energy, (2) the vertical electron affinity, and (3) the six lowest singlet and triplet excitations of the neutral gas-phase benzene molecule.^56^ This procedure yields a fit which is comparable to or better than traditional PPP models^96^ and gives *U*_*nn*_ = 9.69 eV for the on-site repulsion, *t* = 2.70 eV for the nearest-neighbor hopping matrix element, ε = 1.56 eV for the dielectric constant, and *Q* = −0.65 eÅ^2^ for the π-electron quadrupole moment. These values of *U*_*nn*_, *t*, and ε are consistent with those used in previous π-electron models,^95,96^ while *Q* is a new physical parameter in our approach, which takes the place of the ad hoc functional forms assumed in PPP models and governs the corrections to 1/*r* interactions at short distances. These values were used for both molecules considered in this article.

Molecular geometries were obtained by optimizing the isolated molecules using Q-Chem 3.0^97^ with DFT employing the B3LYP functional and 6-311G** basis. The molecules were then chemisorbed (terminal hydrogens removed) to the FCC hollow binding site of an Au(111) surface with the Au–S bond lengths of 2.10 and 2.48 Å in the BDT and CC junctions, respectively.^62,98^

Electrostatically, the electrodes are modeled as metallic spheres with radii of 0.5 nm, and the partially ionic character of the gold–sulfur bond was accounted for by placing point charges of −0.67*e* at the locations of the sulfur atoms.

This value was determined in conjunction with the tunneling-width matrix values, where the average trace of each electrode’s matrix was found to be ≈ 0.44 eV via a simultaneous fit of the experimental thermopower^99^ and conductance.^100^ The same tunnel-coupling values were used for both junctions. Image charge effects reduce the fundamental gap of both junctions and particle-hole symmetry is broken by the formation of S–Au dipoles (see Supporting Information).

### NEGF Theory

We utilize a NEGF theory to describe the quantum transport. In the elastic cotunneling regime, the linear response coefficients may be calculated from the transmission function given by^46^
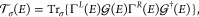
29where the subscript σ indicates a trace over spin, and  and  are the retarded and advanced Green’s functions of the junction, respectively. The tunneling-width matrix for contact α may be expressed as
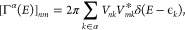
30where *n* and *m* label π-orbitals within the molecule, and *V*_*nk*_ is the coupling matrix element between orbital *n* of the molecule and a single-particle energy eigenstate of energy ϵ_*k*_ in electrode α. We consider transport in the broadband limit, where this matrix can be treated as independent of energy.

In the many-body MDE theory, the Green’s function of a SMJ may be written without approximation as^46^
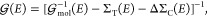
31where  is the molecular Green’s function, Σ_T_ is the tunneling self-energy matrix, whose imaginary part is given by ImΣ_T_ = –*∑*_α_Γ^α^/2, and ΔΣ_C_ is the correction to the Coulomb self-energy due to the broadening of the molecular resonances in the junction. At room temperature and for small bias voltages, ΔΣ_C_ ≈ 0 in the cotunneling regime^46^ (i.e., for nonresonant transport). Furthermore, the inelastic transmission probability is negligible compared with eq 29 in that limit.

The molecular Green’s function  is found by exactly diagonalizing the molecular Hamiltonian, projected onto a basis of relevant atomic orbitals^46^
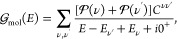
32where *E*_ν_ is the eigenvalue associated with eigenstate ν of the molecular Hamiltonian *H*_mol_,  is the probability that the state ν is occupied, and *C*^νν′^ is a matrix with elements
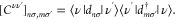
33Here, *d*_*n*σ_ annihilates an electron of spin σ on the *n*th atomic orbital of the molecule. For linear response,  is given by the grand canonical ensemble.

For the single-particle Hückel calculations, the junction Green’s function may be expressed as
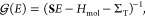
34where *H*_mol_ = *H*^(1)^ is the effective Hückel molecular Hamiltonian and **S** is the overlap matrix which reduces to the identity matrix in an orthonormal basis.

## Supporting Information Available

The influence of tunnel-coupling strength and asymmetry on the thermoelectric response near a node, details regarding the determination of the peak splitting and chemical potentials, details of the Kondo temperature estimation, an analysis of the correspondence between *Z*_s_*T* and η_s_^max^, and an analysis of the influence of many-body correlations on these results are presented here (PDF)
