# Supplementary material for: Quantum Interference Enhancement of the Spin-Dependent Thermoelectric Response
Source: ACS Nano. 2024 Apr 23;18(18):11876–85. doi: 10.1021/acsnano.4c01297 (PMC11080465; doi:10.1021/acsnano.4c01297)
Supplement: Supplementary file 1 — nn4c01297_si_001.pdf [file nn4c01297_si_001.pdf]

Supporting Information: Quantum Interference Enhancement of the Spin-dependent Thermoelectric Response

Runa X. Bennett,† Joshua R. Hendrickson,‡ and Justin P. Bergfield*,†,¶

†*Department of Physics, Illinois State University, Moulton Hall, Normal, IL, 61761, USA*

‡*Air Force Research Laboratory, Sensors Directorate, Wright-Patterson Air Force Base, Ohio, 45433, USA*

¶*Department of Chemistry, Illinois State University, Julian Hall, Normal, IL, 61761, USA*

E-mail: jpbergf@ilstu.edu

Influence of coupling strength and asymmetry near the nodal energy

In the main text we claimed that, away from any molecular resonances, the influence of asymmetry and the total coupling strength on the transmission function was minimal near a node. We show this explicitly using a full many-body calculation of Au-3-methylenepenta-1,4-diyne-1,5-dithiol-Au (CC) and Au-1,3-benzenedithiol-Au (1,3-BDT) junctions, shown schematically in Figs. S1 and S2. The electrodes are modeled as metallic spheres with radii of 0.5 nm and the partially ionic character of the gold-sulfur bond was accounted for by placing point charges of $-0.67e$ at the locations of the sulfur atoms. This value was determined in conjunction with the tunneling-width matrix ($\Gamma^L = \Gamma^R = 0.44eV$) via a simultaneous fit of the experimental thermopower[S1] and conductance.[S2] Image charge and off-resonant orbital

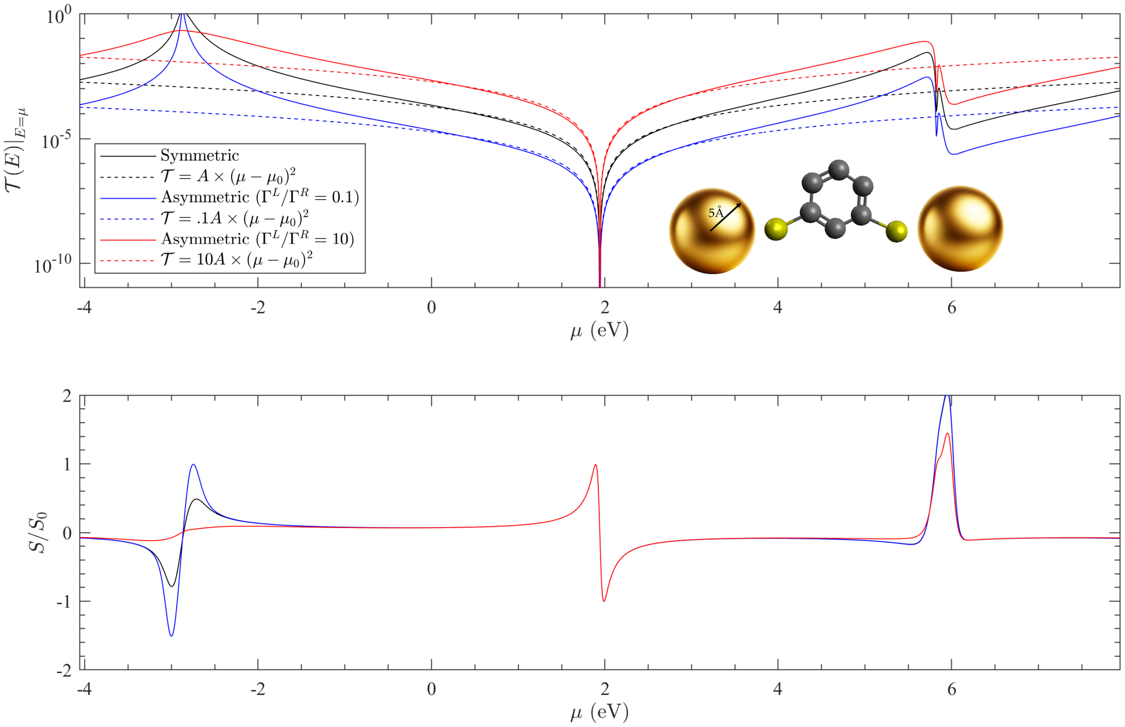

Figure S1: The calculated transmission function (top panel) and thermopower (bottom panel) of a 1,3-BDT junction with different coupling strengths and coupling symmetries. Calculations were performed via exact diagonalization of the effective Hamiltonian derived from first principles. Although the transmission function is not symmetric (the HOMO and LUMO resonances differ significantly due to orbital symmetry, charging, and image charge effects), the transmission near the nodal energy $\mu_0 \approx 1.94$eV is nearly quadratic (fits are shown in the dashed lines). Consequently, the thermopower near μ_0 is nearly identical in all cases, despite the wide range of coupling strength and asymmetry values. In these calculations the constant $A = 5 \times 10^{-5}$ and the temperature was set to 300K.

terms are included leading to the following renormalized parameters: An on-site repulsion of $U_{nn} = 9.69eV$, an effective inter-molecular hopping integral $t = 2.70eV$, an on-site energy of $\varepsilon_n = -4.06eV$, effective π quadrupole moment of $Q = -0.64e\text{Å}^2$ and a dielectric screening of $\varepsilon = 1.56$.[S3]

Molecular geometries were obtained by optimizing the isolated molecules using Q-Chem 3.0[S4] with density functional theory employing the B3LYP functional and 6-311G** basis. The molecules were then chemisorbed (terminal hydrogens removed) to the FCC hollow binding site of a Au(111) surface with the Au–S bond lengths of 2.10Å and 2.48Å in the

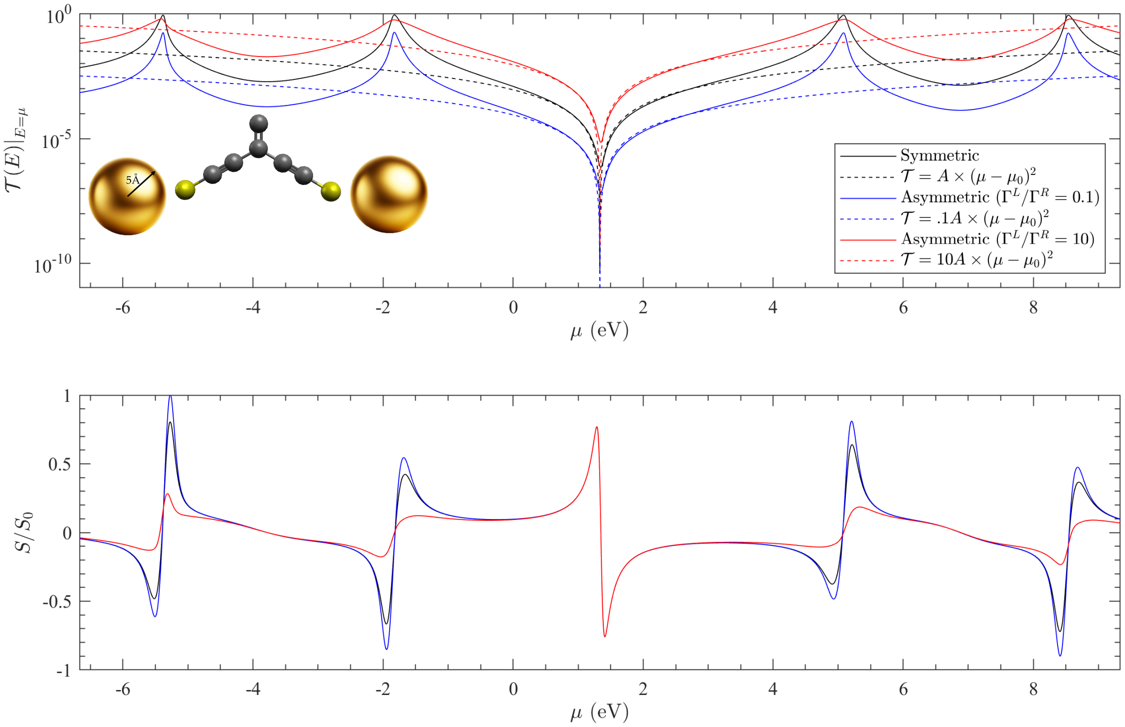

Figure S2: The calculated transmission function (top panel) and thermopower (bottom panel) of a CC junction with different coupling strengths and coupling symmetries. Calculations were performed via exact diagonalization of the effective Hamiltonian derived from first principles including thiol end-groups, image charge effects, etc. Like the 1,3-BDT junction these spectra are not symmetric. Near the nodal energy ($\mu_0 \approx 1.33$eV) the transmission is nearly quadratic (see fits shown in the dashed lines). Consequently, the thermopower in each case are indistinguishable when $\mu \sim \mu_0$. For the quadratic fit $A = 5 \times 10^{-4}$. The temperature was set to 300K.

BDT and CC junctions, respectively.[S5,S6] Image charge effects reduce the fundamental gap of each molecule significantly.

As indicated in Figs. S1 and S2, variations in the tunnel-coupling symmetry and total strength of several orders of magnitude play a negligible role on the form of the transport near the nodal energy, i.e. $\mathcal{T}(E) \propto (E - \mu_0)^2$, where $\mu_0 \approx 1.94$eV and ≈ 1.333eV in the BDT and CC junctions, respectively. Comparing these results to the Hückel calculations in Fig. 1 of the manuscript shows that electron-electrons also don't influence the form of $\mathcal{T}$ and therefore S near the node. These conclusions are supported by alternative theoretical

methods[S6–S12] and direct experimental observations[S13–S16] of similar systems.

The near-universality of the thermopower near a node detuned from any molecular resonances can also be understood by realizing that the dominant contribution to the transport in the mid-gap region are the tails of the HOMO and LUMO resonances. In junctions with a node, the junction's Green's function in that case gives a transmission which tends to zero quadratically in that case.[S13,S15,S17]

The Kondo temperature

The Kondo effect involves correlations between the lead and molecular electrons, leading to screening of unpaired spins within the junction at temperatures below the Kondo temperature T_K. The Kondo temperature in a SMJ may be estimated from the formula[S18–S20]

$$k_B T_K \sim \frac{1}{2}\sqrt{\tilde{\Gamma} U} \exp\left(-\frac{\pi(\mu - \varepsilon_{\text{HOMO}})(\varepsilon_{\text{LUMO}} - \mu)}{\tilde{\Gamma} U}\right), \tag{1}$$

where $\mu \approx \mu_0$, $\tilde{\Gamma} = \sum_\alpha \tilde{\Gamma}^\alpha$ is the total effective tunnel-coupling strength, $U = \varepsilon_{\text{LUMO}} - \varepsilon_{\text{HOMO}}$ is the HOMO-LUMO gap and μ is the chemical potential. $\tilde{\Gamma}^\alpha$ is not equal to $\sum_\alpha \Gamma^\alpha$ since the many-body matrix elements are not equal to unity. We extract $\tilde{\Gamma}$ from the transmission function where $\tilde{\Gamma}$ is the full-width at half maximum (FWHM).

We can estimate the Kondo temperature for the 1,3-BDT junction, using the nodal energy $\mu \approx 1.94$eV, $U \approx 8.58$eV, $\mu - \varepsilon_{\text{HOMO}}$=1.94+2.87eV, $\mu + \varepsilon_{\text{HOMO}}$=5.71-1.94eV, and $\tilde{\Gamma} \approx 2 \times .1569$eV, giving $T_K \approx 5.99 \times 10^{-6}$K. We have assumed the same FWHM for both the HOMO and LUMO resonance in the 1,3-BDT junction since the LUMO resonance deviates strongly from a Lorentzian in this case. For the CC junction, $\mu \approx 1.33$eV, $U \approx 6.9$eV, $\mu - \varepsilon_{\text{HOMO}}$=1.33+1.82eV, $\mu + \varepsilon_{\text{HOMO}}$=5.08-1.33eV, and $\tilde{\Gamma} \approx 0.1882 + 0.1255$eV, giving $T_K \approx 2.98 \times 10^{-4}$K. These calculations support our claim that the T_K is below the mK range. Molecules containing magnetic elements are more favorable for the observation of the Kondo effect.[S21,S22]

Peak splitting and chemical potential for $Z^{\text{el}}_{(\text{s})}T$

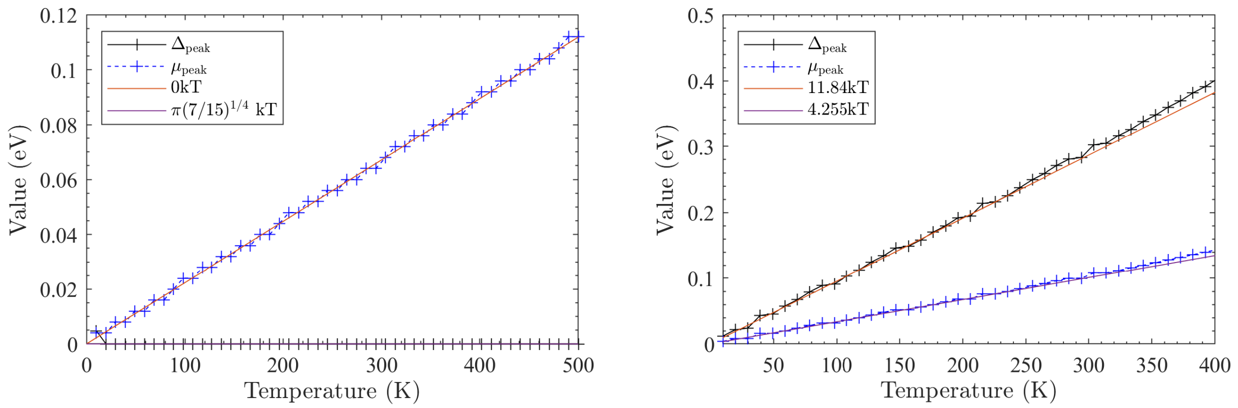

(a) ZT (b) $Z_\text{s}T$

Figure S3: The calculated spin-splitting Δ_{peak} and chemical potential μ_{peak} needed to realize peak performance for (a) ZT and (b) $Z_\text{s}T$ as a function of temperature. The analytic fit for ZT's peak values and numerical fit for $Z_\text{s}T$'s peak values are shown in teh solid lines of each panel. Calculations were for the 1,3-BDT junction although the results apply to any quadratic node.

The total and spin figures-of-merit, which may be expressed as[S24,S25]

$$Z_{(\text{s})}T = \frac{S^2_{(\text{s})}|G_{(\text{s})}|T}{\kappa}, \tag{2}$$

has a peak value which differs from S_s since G_s and κ are functions of electron energy and temperature. Although analytic solutions for the spin-splitting and chemical potential needed for peak performance, i.e. Δ_{peak} and μ_{peak}, are possible for simple quadratic nodes they are complex expressions. Here, we extract the peak values numerically from the calculated response of a BDT junction, where we vary temperature, Δ, and μ using Hückel+NEGF theory.

We include analyses for both ZT and $Z_\text{s}T$, shown in Fig. S4c and Fig. S3b, respectively. As discussion in the main text, according to theory ZT should be peaked when $\Delta = 0$ and $\mu = \pi(7/15)^{1/4}kT$. The calculated Δ_{peak} and μ_{peak} values shown in panel (a) of the figure support these theoretical results and show our method can accurately extract peak value

quantities from the data. Applying this procedure to $Z_{\rm s}T$, shown in panel (b) of the figure, we find the values reported in the main text.

The influence of interactions on the spin-thermoelectric response

In this section, we repeat the calculations used to generate Fig.2 and 3 of the manuscript using an effective Hückel calculation This method utilizes the optimized geometries, renormalized Hamiltonian, etc. but sets $U_{nm} = 0$. As shown in Fig. S4, $S_{\rm (s)}$, $Z_{\rm (s)}$ and $\eta^{\rm max}_{\rm (s)}$ are all identical to the many-body case, as expected since the transmission function's *form* is nearly universal. As in the many-body case, $\max(S_s) = 2S_0$ when $\mu = 0$ and $\Delta = \Delta^{\rm max}_{S_s}$, where

$$\Delta^{\rm max}_{S_s} = \frac{2\pi}{\sqrt{3}} kT \approx 3.63kT. \tag{3}$$

Similarly, $Z^{\rm el}T$ and $\eta^{\rm max}$ decrease monotonically as a function of Δ while $Z^{\rm el}_{\rm s}T$ and $\eta^{\rm max}_{\rm s}$ are peaked, as indicated in sub-figures (c) and (d), respectively. $Z^{\rm el}_{\rm s}T$ reaches a maximum value of 1.51 while $\eta^{\rm max}_{\rm s}$ reaches 27.96% of Carnot when $\Delta = \Delta^{\rm max}_{Z_{\rm s}T}$.

In contrast, the influence of $\kappa_{\rm alt}$, shown in Fig. S5 is different when Hückel or many-body theory is used. Comparing the left- and right-hand panels of the figure reveals that for the same $\kappa_{\rm alt}$, the peak values of $Z_{\rm s}T$ are reduced (proportionally) less than those of ZT. For the systems considered here, this occurs because $Z_{\rm s}T$ peaks when $\Delta > 0$ which *increases* $\kappa_{\rm el}$ and reduces $\kappa_{\rm alt}/\kappa_{\rm el}$. For instance, when $\kappa_{\rm alt} = 10^{-6}\kappa_0$ and Δ is fixed (top panels), the peak value of $Z_{\rm s}T$ is reduced by 14.8% while the peak value of ZT is reduced by 44.9%.

When Δ is tuned, the reductions generally decrease and the spectral width of the enhancement increases. For the same $\kappa_{\rm alt}$ value with tuned Δ (bottom panels), the reduction of the peak value of $Z_{\rm s}T$ becomes 11.5% while ZT remains unchanged, although in both cases the enhancement has broadened significantly in μ. As $\kappa_{\rm alt}$ is increased so does the

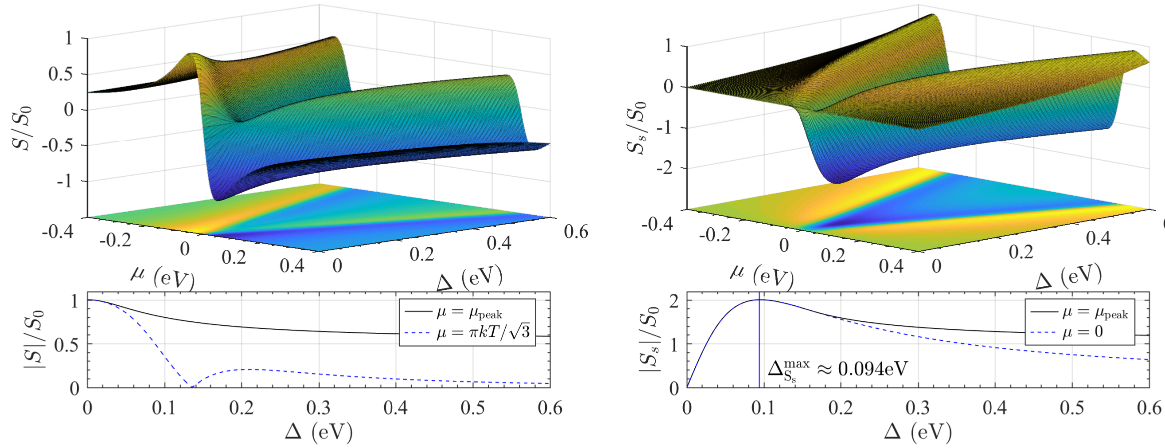

(a) Charge thermopower (S)

(b) Spin-thermopower (S_s)

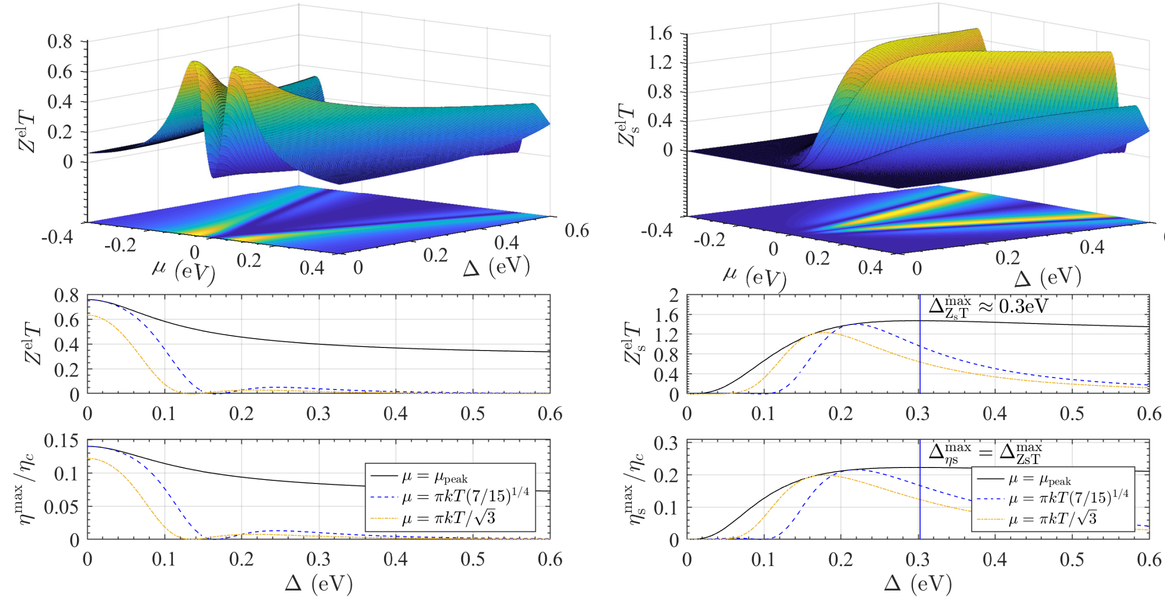

(c) Charge figure-of-merit ($Z^{\text{el}}T$) and $\eta^{\max}$

(d) Spin figure-of-merit ($Z^{\text{el}}_{\text{s}}T$) and $\eta^{\max}_{\text{s}}$

Figure S4: The calculated (a) charge thermopower S, (b) spin-thermopower S_{s}, (c) $Z^{\text{el}}T$, $\eta^{\max}$ and (d) spin $Z^{\text{el}}_{\text{s}}T$, $\eta^{\max}_{\text{s}}$ spectra of a 1,3-BDT junction's π-system shown as functions of electrode chemical potential μ and spin-splitting $\Delta = 2g\mu_B B$ using Hückel+NEGF theory. Charge quantities decrease monotonically with increasing Δ. Spin quantities are peaked, reaching maxima when $\Delta = 2\pi/\sqrt{3}kT \approx 3.63kT$ and $\Delta \approx 11.8kT$ for S_{s} and $Z_{\text{s}}T$ ($\eta^{\max}_{\text{s}}$), respectively. In the lower panels quantities are shown with μ tuned to give the maximum response ($\mu = \mu_{\text{peak}}$) and with μ fixed to specific values. Calculations are for $T = 300K$ such that $S_0 = \pi/\sqrt{3}(k/e) \approx 156\mu$V/K.

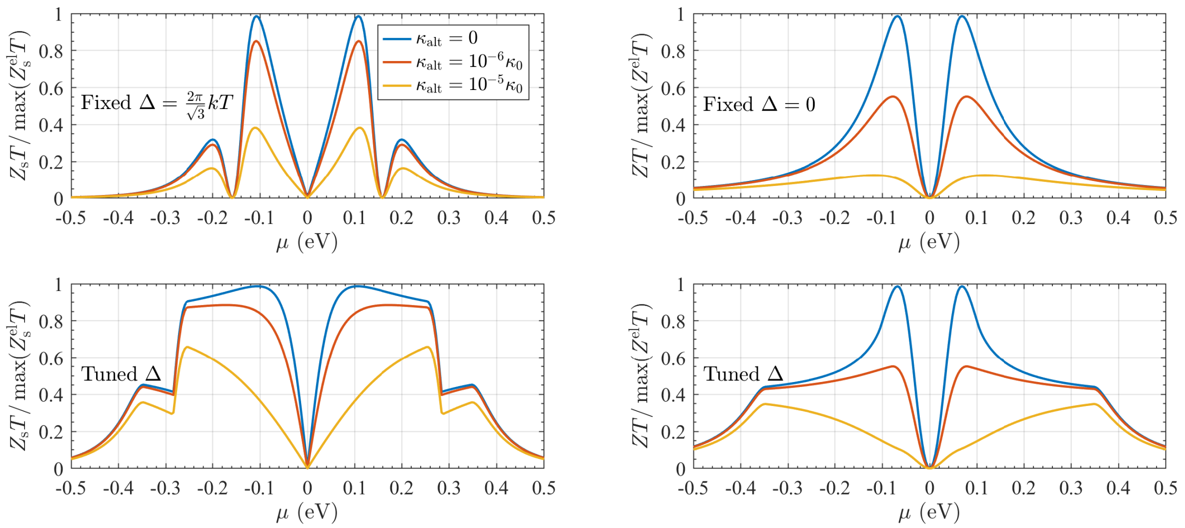

Figure S5: The influence of $\kappa_{\rm alt}$ on the $Z_{\rm s}T$ (left panels) and ZT (right panels) spectra of a 1,3-BDT junction calculated using Hückel+NEGF theory. Values are normalized to the node-possessing channel's peak value. Owing to the influence of Δ on κ, $Z_{\rm s}T$ is less sensitive to additional thermal channels than ZT. When $\kappa_{\rm alt} = 10^{-6}\kappa_0$, $Z_{\rm s}T$ is reduced by 11.5% when Δ is tuned and 14.8% when Δ is fixed while ZT is reduced by 44.9% in both cases. The optimal Δ is a function of the total transport, so the importance of tuning Δ increases with $\kappa_{\rm alt}$. When $\kappa_{\rm alt} = 10^{-5}\kappa_0$, $Z_{\rm s}T$ is reduced by 34.2% and 61.7% when Δ is tuned or fixed, respectively, while ZT is reduced by 65.2% and 87.5%, respectively. $\kappa_0 = \pi^2 k^2 T/3h \sim$ 289pW/K at $T = 300K$.

importance of tuning Δ. With $\kappa_{\rm alt} = 10^{-5}\kappa_0$, $Z_{\rm s}T$ is reduced by 61.7% when Δ is fixed but only 34.2% when Δ is tuned. Similarly, ZT is reduced by 87% when Δ is fixed and 65% when Δ is tuned for the same $\kappa_{\rm alt}$. Although the values are different for the Hückel calculations, the trends and relative influence of $\kappa_{\rm alt}$ on the spin-response are very similar to the many-body case presented in the main text.

Relationship between the $\eta_{(\rm s)}^{\rm max}$ and $Z_{(\rm s)}T$ spectra and the importance of interactions

The calculated ZT and $\eta^{\rm max}$ spectra and $Z_{\rm s}T$ and $\eta_{\rm s}^{\rm max}$ spectra of a 1,3-BDT junction are shown in panels (a) and (b) of Fig. S6, respectively, as a function of electrode chemical

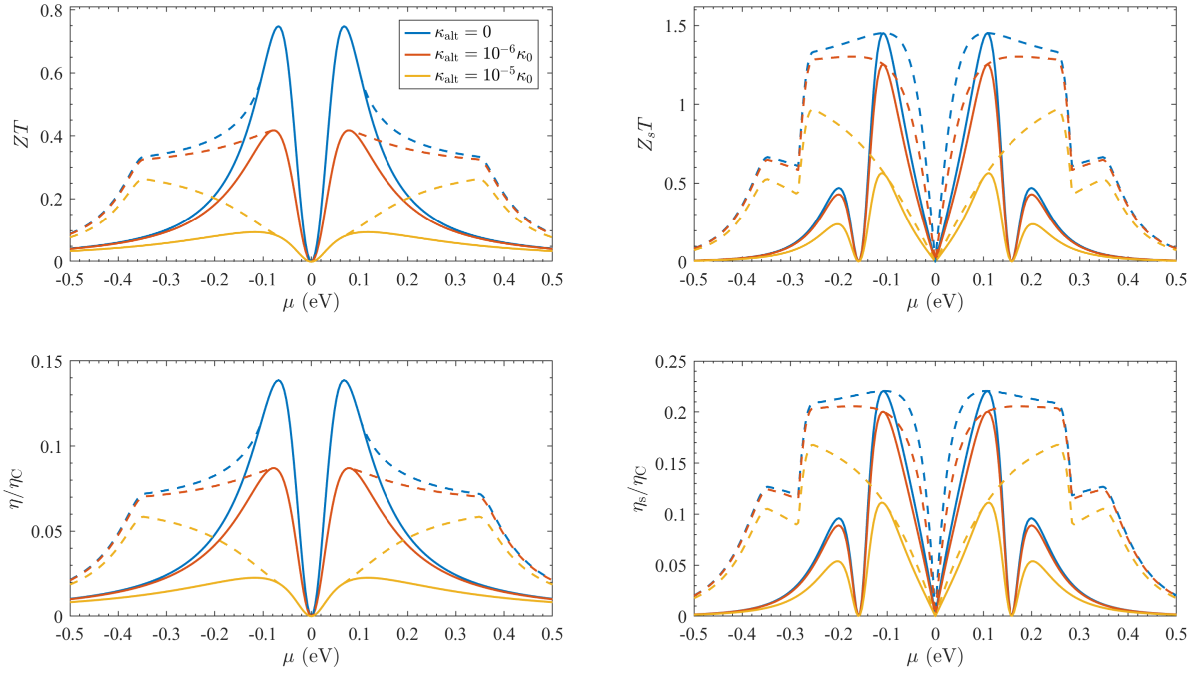

(a) Total charge ZT and η^{max}

(b) Spin Z_sT and η_s^{max}

Figure S6: The calculated (a) ZT and η^{max} spectra and (b) Z_sT and η_s^{max} spectra of a 1,3-BDT junction as a function of electrode chemical potential μ. The nodal energy is set to zero for convenience. Dashed lines and solid lines represent fixed spin-splittings, respectively, with $\Delta = 0$ for the total charge quantities and $\Delta = 2\pi/\sqrt{3}kT$ for the spin quantities. Variations in $Z_{(s)}T$ match variations $\eta_{(s)}^{max}$, indicating that ZT is a good measure of maximum thermodynamic performance in these system. Calculations utilize many-body theory with π-EFT and are for junctions operating at 300K.

potential μ. Calculations were performed using MDE many-body theory with π-EFT with the nodal energy is set to zero for convenience. Solid lines correspond to $\Delta = 0$ in the ZT and η^{max} plots and $\Delta = 2\pi/\sqrt{3}kT$ in the Z_sT and η_s^{max} plots. In both the fixed and tuned cases, variation in $Z_{(s)}T$ match variations in $\eta_{(s)})^{max}$, supporting our claim that the figure-of-merit is an accurate measure of the maximum thermodynamic device performance in these systems.[S23] For example, with $\kappa_{alt} = 10^{-6}\kappa_0$ and Δ fixed, ZT is reduced by 73.5% and η^{max} by 67.4% while Z_sT is reduced by 37.4% and η_s^{max} by 27.3%.

As usual, the reduction in these quantities can be mitigated when Δ is tuned. For $\kappa_{alt} = 10^{-6}\kappa_0$ this gives ZT and η^{max} reductions of 60.6% and 53.5%, respectively, with significantly

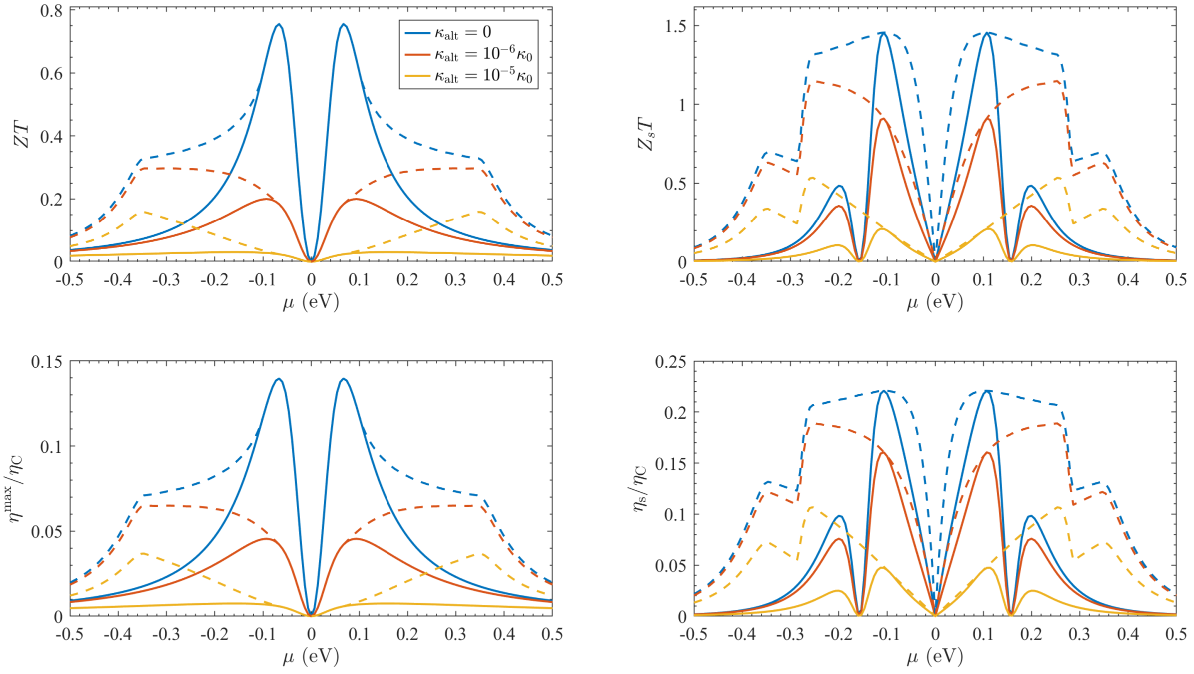

(a) Total charge ZT and $\eta^{\rm max}$ (b) Spin $Z_{\rm s}T$ and $\eta_{\rm s}^{\rm max}$

Figure S7: The calculated (a) ZT and $\eta^{\rm max}$ spectra and (b) $Z_{\rm s}T$ and $\eta_{\rm s}^{\rm max}$ spectra of a 1,3-BDT junction using Hückel+NEGF theory. The nodal energy is set to zero for convenience. Dashed lines and solid lines represent fixed spin-splittings, respectively, with $\Delta = 0$ for the total charge quantities and $\Delta = 2\pi/\sqrt{3}kT$ for the spin quantities. Although the trends are still essentially the same as in the many-body case, the reductions are slightly different (cf Tab. S2). Calculations are for junctions operating at 300K.

Table S1: Influence of $\kappa_{\rm alt}$ on $Z_{\rm (s)}T$ and $\eta_{\rm (s)}^{\rm max}$ from many-body theory.

Quantity	Δ	Reduction with $\kappa_{\rm alt} = 10^{-6}\kappa_0$	Reduction with $\kappa_{\rm alt} = 10^{-5}\kappa_0$
ZT	Fixed	73.5%	95.9%
$\eta^{\rm max}$	Fixed	67.4%	94.6%
$Z_{\rm s}T$	Fixed	37.4%	85.6%
$\eta_{\rm s}^{\rm max}$	Fixed	27.3%	78.5%
ZT	Tuned	60.6%	79%
$\eta^{\rm max}$	Tuned	53.4%	73.7%
$Z_{\rm s}T$	Tuned	21.1%	63.3%
$\eta_{\rm s}^{\rm max}$	Tuned	14.4%	51.7%

broader responses. The spin-response sees similar changes with $Z_{\rm s}T$ and $\eta_{\rm s}^{\rm max}$ exhibiting reductions of only 21.1% and 14.5%, respectively. A full accounting for the reductions found

can be found in Tab. S1

Table S2: Influence of κ_{alt} on $Z_{(\text{s})}T$ and $\eta^{\text{max}}_{(\text{s})}$ from Hückel+NEGF theory.

Quantity	Δ	Reduction with $\kappa_{\text{alt}} = 10^{-6}\kappa_0$	Reduction with $\kappa_{\text{alt}} = 10^{-5}\kappa_0$
ZT	Fixed	44.14%	87.3%
η^{max}	Fixed	37.2%	83.7%
$Z_{\text{s}}T$	Fixed	13.7%	61.2%
$\eta^{\text{max}}_{\text{s}}$	Fixed	9.14%	49.6%
ZT	Tuned	44.4%	64.7%
η^{max}	Tuned	37.2%	57.9%
$Z_{\text{s}}T$	Tuned	10.28%	33.3%
$\eta^{\text{max}}_{\text{s}}$	Tuned	6.8%	24%

Unlike the nodal response, interaction play a role in determining the influence of κ_{alt}. This occurs because the magnitude of the thermal conductance is roughly proportional to the transmission which *does* depend strongly on the chemical structure, energy levels, etc. The response for the same junction found using Hückel+NEGF (i.e. MDE many-body theory with $U_{nm} = 0$) is shown in Fig. S7. The reduction values in this case are given in Tab. S2, where smaller reductions are exhibited since the transmission predicted by Hückel+NEGF theory is significantly larger than that predicted by many-body theory.

References

(S1) Baheti, K.; Malen, J.; Doak, P.; Reddy, P.; Jang, S.-Y.; Tilley, T.; Majumdar, A.; Segalman, R. Probing the Chemistry of Molecular Heterojunctions Using Thermoelectricity. *Nano Lett.* **2008**, *8*, 715–719.

(S2) Xiao, X.; Xu, B.; Tao, N. Measurement of Single Molecule Conductance: Benzenedithiol and Benzenedimethanethiol. *Nano Lett.* **2004**, *4*, 267–271.

(S3) Barr, J. D.; Stafford, C. A.; Bergfield, J. P. Effective field theory of interacting π electrons. *Phys. Rev. B* **2012**, *86*, 115403.

(S4) Shao, Y.; Molnar, L. F.; Jung, Y.; Kussmann, J.; Ochsenfeld, C.; Brown, S. T.; Gilbert, A. T.; Slipchenko, L. V.; Levchenko, S. V.; O'Neill, D. P.; others Advances in methods and algorithms in a modern quantum chemistry program package. *Phys. Chem. Chem. Phys.* **2006**, *8*, 3172–3191.

(S5) Bilić, A.; Reimers, J. R.; Hush, N. S. The structure, energetics, and nature of the chemical bonding of phenylthiol adsorbed on the Au (111) surface: Implications for density-functional calculations of molecular-electronic conduction. *J. Chem. Phys.* **2005**, *122*.

(S6) Solomon, G. C.; Bergfield, J. P.; Stafford, C. A.; Ratner, M. A. When "small" terms matter: Coupled interference features in the transport properties of cross-conjugated molecules. *Beilstein J. Nanotechnol.* **2011**, *2*, 862–871.

(S7) Pedersen, K. G.; Strange, M.; Leijnse, M.; Hedegård, P.; Solomon, G. C.; Paaske, J. Quantum interference in off-resonant transport through single molecules. *Phys. Rev. B* **2014**, *90*, 125413.

(S8) Pedersen, K. G.; Borges, A.; Hedegaå rd, P.; Solomon, G. C.; Strange, M. Illusory connection between cross-conjugation and quantum interference. *J. Phys. Chem. C* **2015**, *119*, 26919–26924.

(S9) Markussen, T.; Stadler, R.; Thygesen, K. S. The relation between structure and quantum interference in single molecule junctions. *Nano Lett.* **2010**, *10*, 4260–4265.

(S10) Solomon, G. C.; Andrews, D. Q.; Goldsmith, R. H.; Hansen, T.; Wasielewski, M. R.; Van Duyne, R. P.; Ratner, M. A. Quantum Interference in Acyclic Systems: Conductance of Cross-Conjugated Molecules. *J. Am. Chem. Soc.* **2008**, *130*, 17301–17308.

(S11) Andrews, D. Q.; Solomon, G. C.; Goldsmith, R. H.; Hansen, T.; Wasielewski, M. R.; Duyne, R. P. V.; Ratner, M. A. Quantum interference: The structural dependence of electron transmission through model systems and cross-conjugated molecules. *J. Phys. Chem. C* **2008**, *112*, 16991–16998.

(S12) Solomon, G. C.; Andrews, D. Q.; Van Duyne, R. P.; Ratner, M. A. Electron transport through conjugated molecules: When the π system only tells part of the story. **2009**, *10*, 257–264.

(S13) Liu, J.; Huang, X.; Wang, F.; Hong, W. Quantum interference effects in charge transport through single-molecule junctions: detection, manipulation, and application. *Acc. Chem. Res.* **2018**, *52*, 151–160.

(S14) Miao, R.; Xu, H.; Skripnik, M.; Cui, L.; Wang, K.; Pedersen, K. G.; Leijnse, M.; Pauly, F.; Waarnmark, K.; Meyhofer, E.; others Influence of quantum interference on the thermoelectric properties of molecular junctions. *Nano Lett.* **2018**, *18*, 5666–5672.

(S15) Li, Y.; Buerkle, M.; Li, G.; Rostamian, A.; Wang, H.; Wang, Z.; Bowler, D. R.; Miyazaki, T.; Xiang, L.; Asai, Y.; others Gate controlling of quantum interference and direct observation of anti-resonances in single molecule charge transport. *Nat. Mater.* **2019**, *18*, 357–363.

(S16) Guédon, C. M.; Valkenier, H.; Markussen, T.; Thygesen, K. S.; Hummelen, J. C.; van der Molen, S. J. Observation of quantum interference in molecular charge transport. *Nat. Nanotechnol.* **2012**, *7*, 305–309.

(S17) Bergfield, J.; Stafford, C. A. Thermoelectric signatures of coherent transport in single-molecule heterojunctions. *Nano Lett.* **2009**, *9*, 3072–3076.

(S18) Pustilnik, M.; Glazman, L. Kondo effect in quantum dots. *J. Phys. Condens. Mat.* **2004**, *16*, R513–r537.

(S19) Thygesen, K. S.; Rubio, A. Nonequilibrium GW approach to quantum transport in nano-scale contacts. *J. Chem. Phys.* **2007**, *126*, 091101.

(S20) Haldane, F. Scaling theory of the asymmetric Anderson model. *Phys. Rev. Lett.* **1978**, *40*, 416.

(S21) Liang, W.; Shores, M. P.; Bockrath, M.; Long, J. R.; Park, H. Kondo resonance in a single-molecule transistor. *Nature* **2002**, *417*, 725–729.

(S22) Yu, L. H.; Keane, Z. K.; Ciszek, J. W.; Cheng, L.; Tour, J. M.; Baruah, T.; Pederson, M. R.; Natelson, D. Kondo Resonances and Anomalous Gate Dependence in the Electrical Conductivity of Single-Molecule Transistors. *Phys. Rev. Lett.* **2005**, *95*.

(S23) Bergfield, J. P.; Solis, M. A.; Stafford, C. A. Giant thermoelectric effect from transmission supernodes. *ACS Nano* **2010**, *4*, 5314–5320.

(S24) Benenti, G.; Casati, G.; Saito, K.; Whitney, R. S. Fundamental aspects of steady-state conversion of heat to work at the nanoscale. *Phys. Rep.* **2017**, *694*, 1–124.

(S25) DiSalvo, F. J. Thermoelectric cooling and power generation. *Science* **1999**, *285*, 703–706.
